# Challenges of middle-aged men in utilizing new health services from primary health care providers' perspective: a qualitative study

**DOI:** 10.1186/s12875-022-01933-2

**Published:** 2022-12-07

**Authors:** Nayeb Fadaei Dehcheshmeh, Seyed Mehdi Emamian Fard, Tayebeh Roghani, Parvin Mohammadi, Farzad Faraji-Khiavi

**Affiliations:** 1 Department of Public Health, Shoushtar Faculty of Medical Sciences, Shoushtar, Iran; 2 Shoushtar Faculty of Medical Sciences, Shoushtar, Iran; 3 Department of Midwifery, School of Nursing and Midwifery, Ahvaz Jundishapur University of Medical Sciences, Ahvaz, Iran; 4 Social Determinants of Health Research Center, Ahvaz Jundishapur University of Medical Sciences, Ahvaz, Iran; 5 Department of Health Services Management, School of Public Health, Ahvaz Jundishapur University of Medical Sciences, Ahvaz, Iran

**Keywords:** Utilization, Access, Coverage, Middle-aged men, Community health workers, Primary health care, Health services

## Abstract

**Background:**

Despite its special importance among different age groups, the middle-aged male group has often been neglected in the Iranian health system. The aim of this study was to examine, from the perspective of primary health care providers, the challenges of middle-aged men in utilizing health services.

**Methods:**

This is a qualitative research that was conducted using semi-structured interviews in 2020 in Shoushtar Faculty of Medical Sciences, Iran. The research population included 60 managers and staff of the health sector. To collect the data, a group discussion method was used based on purposive sampling method. Data analysis was done manually using the conventional content analysis method with data reduction. Lincoln & Guba’s four criteria of credibility, dependability, confirmability, and transferability were used to assess the trustworthiness of the results.

**Results:**

The challenges of middle-aged men to receive modern health services were identified in 35 codes, 9 categories and three main themes. These themes included Context, Content, and Process. The Context theme comprised the following three categories: personal, economic and sociocultural, and geographic factors. The Content theme contained two categories of staff and facilities. Finally, the Process theme included four categories of service quality, program management, system of information registration and follow-up, and health education and publicizing.

**Conclusion:**

Promoting middle-aged men’s benefits from modern health services calls for overcoming three categories of challenges related to: context, content, and process. Time and place restrictions on access to services should be alleviated by empowering health care workers, improving their working conditions, and strengthening the facilities of comprehensive health service centers. In addition, with proper management of the family physician program and service provision at different levels, the coverage of services for middle-aged men can also be extended.

**Supplementary Information:**

The online version contains supplementary material available at 10.1186/s12875-022-01933-2.

## Background

Primary health care is an essential part of the health system and plays a pivotal role in the economic and social development of countries. Therefore, providing primary health care has a direct impact on the health of people in the community and reduces the number of unnecessary referrals to emergency centers and hospitals [1]. Due to the epidemiological transition, the health system faces challenges in defining high-risk groups using health services and in defining different dimensions of health.

Studies show that gender is one of the most important determinants of disease and plays a role in the pattern of disease development and prevention [2]. According to the 2016 report of the World Health Organization, life expectancy in Iran is 77 years for women and 75 years for men [3]. Biological and clinical factors and low use of health services lead to lower life expectancy in men [4–6]. According to studies, the burden caused by traffic accidents in men is about 5 times that in women, and the burden due to falls in men was 3 times more than that in women [7]. Also, suicide attempts are four times more successful in men than women [8]. Men have different views on health and its priority in life compared to women, so men are still lagging behind women as far as using health care is concerned [4, 9].

Various studies show that most people living with AIDS in Iran are men, and smoking and alcohol consumption is more widespread among men than women [10–12]. Asghari et al. also found that Iranian men experience a higher incidence of diseases compared to women [13]. The relative frequency of smoking in men has been reported to be more than that in women [14]. Studies show that unhealthy lifestyles play a significant role in predisposing people to non-communicable diseases. Behavioral risk factors seem to increase male mortality extensively [9, 15, 16].

In Iran, middle-aged men are considered one of the main pillars of the family, and this age group constitutes half of the active population [10, 15]. Despite its special importance among different age groups, the middle-aged male group has often been neglected in the Iranian health system [17, 18]. Interventions to reduce the burden of disease in this age group do not simply include the control of infectious diseases. Rather, they strongly depend on cultural, social and lifestyle issues [17, 19]. In this regard, in order to increase access to and justice in health services, help people against health costs, improve the quality of services, Iran’s Health Transformation Plan was started in 2014 in inpatient departments and then extended to the field of health [20]. Following the implementation of this plan in the field of health, the middle-aged comprehensive health service program targeting the age group of 30 to 59 years was started offering a series of new health services with the financial support of the Ministry of Health. These services were for diseases such as diabetes, obesity, hypertension, dyslipidemia, mental disorders, etc. [17]. The results of a study by Emamgholipour show that 50% of deaths in people aged 15–49 years in Iran are due to unhealthy lifestyles including diet, physical activity and smoking [21].

Men procrastinate going to medical centers for various reasons until advanced and dangerous stages of the disease [22]. Various factors affect the rate of clients’ referral and non-referral to health service centers [23]. Although more susceptible to certain types of diseases, men do not tend to use primary health care, and studies have shown that compared with women, men are less likely to refer to care systems for receiving care [4, 22, 24]. In addition, the referral of middle-aged men to health centers in Iran is not based on a suitable approach, and the referral index of this age group for receiving basic health care is also low [25]. Statistics show that more than 7 years after the implementation of the health transformation plan in Iran, only 18% of middle-aged men in Shoushtar city have received modern health services. According to the above, one of the research priorities in this region is service coverage for middle-aged men. However, no study has yet been done on this very topic based on our best knowledge. Therefore, the aim of this study was to investigate, from the perspective of primary health care providers, the challenges of middle-aged men in utilizing health services.

## Methods

### Study design and setting

This qualitative research was conducted using the semi-structured interview method in 2020. Iran’s health system is organized at three levels: national, provincial, and county [26]. The management and organization of services is centralized within the government and is carried out by the Ministry of Health, Treatment and Medical Education [27]. Universities operate independently but under the general laws and policies of the Ministry of Health and Medical Education. At the provincial level, universities of medical sciences are in charge of primary health services in the health sector. The health and treatment network of the city is considered as the smallest independent unit of the country’s health and treatment system, and it is considered the executive system of providing health care services. Healthcare services in Iran are provided at three levels. Level one includes the provision of primary health care by county health centers, including urban and rural health centers (CHSCs), health bases (HBs), and health posts (HPs). Specialty and sub-specialty services at secondary and tertiary levels are provided by hospitals and other medical centers [28, 29]. The structure of the health network in Shoushtar city is an example of the structure of the health system in Iran.

**Fig. 1:**
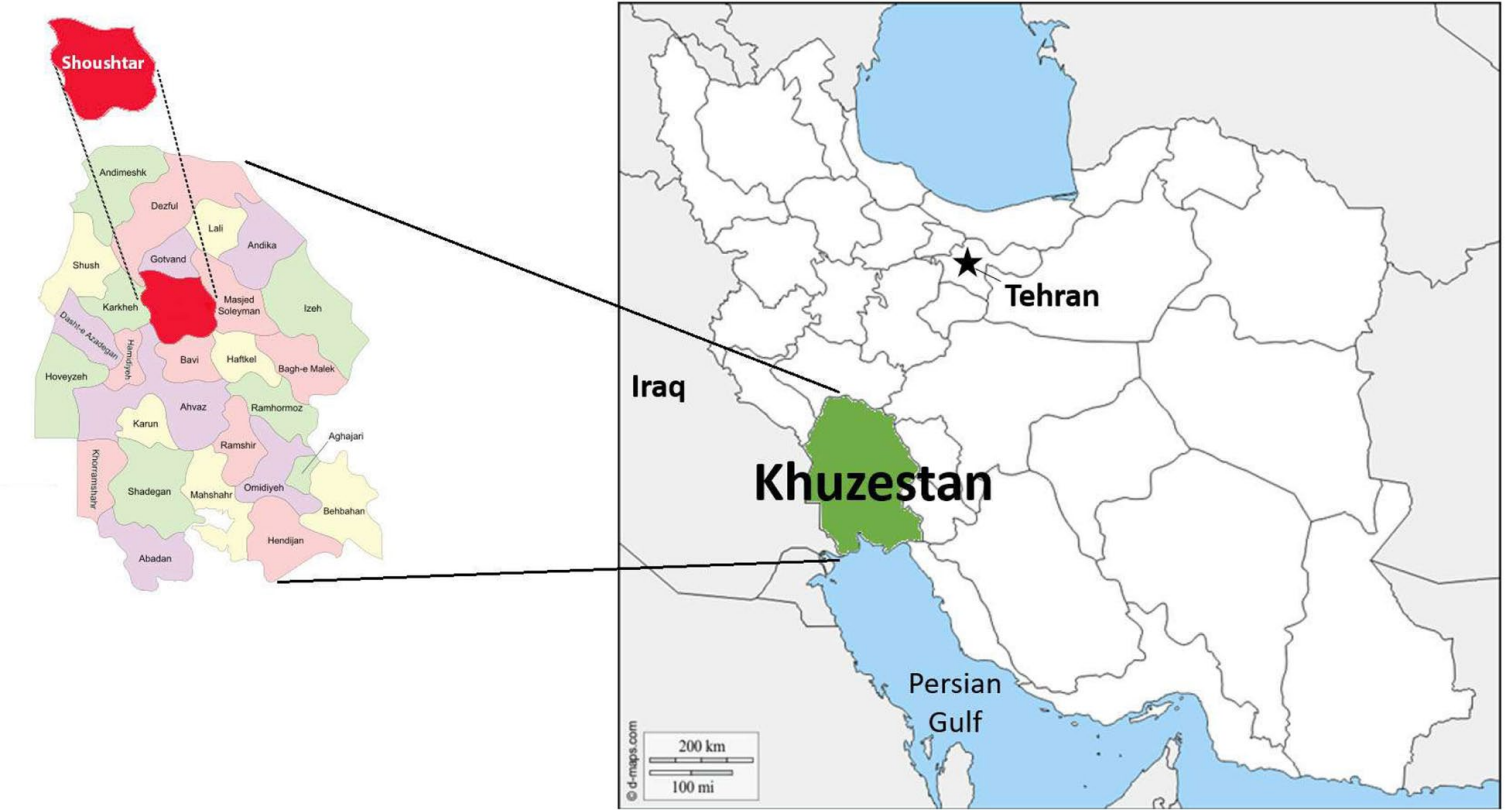
Shoushtar city located in Khuzestan province in the southwest of Iran

This study was conducted in Shoushtar Faculty of Medical Sciences and Health Services located in Khuzestan province in the southwest of Iran (Fig. 1). With a population of nearly 200 thousand people, Shoushtar city is located in the north of Khuzestan province. Men make up more than 50% of the population, of whom 45,714 people (more than 45% of the male population) are middle-aged. Only 18% of these people have received modern health services so far. It should be noted that almost 44% of middle-aged men in Shoushtar are overweight, 58% of them do unfavorable physical activities, and 2% have high blood pressure and diabetes. After April 2014 and with the implementation of the health transformation plan in Iran, the topic of middle-aged men’s health was regarded as the first level of service provision. However, after more than 7 years of the implementation of the program, these middle-aged men have not yet benefited optimally from primary care, and this problem is one of the research priorities in this region.

### Recruitment of participants

The study population consisted of all key informants, experts, and managers, and the staff working at health centers, health bases, and health posts affiliated to Shoushtar University of Medical Sciences. These individuals had sufficient experience in different levels of the health system in addition to having knowledge in delivery of services and care for middle-aged men. Inclusion criteria were having at least 3 years of experience in implementing the men’s health program and giving consent to participate in the study. Participants were excluded from the study if they were unable or unwilling to participate in the study. Purposeful sampling method was used to select the participants. This means that the selected people were those provided the most and richest information to the research team [30]. In order to obtain more information about the subject under study, maximum diversity was maintained in the selection of participants, and people from different groups of service providers who had different views and opinions participated in the group discussion sessions. In this research, data collection continued until data saturation. All sessions were led by two members of the research team (SME and TR).

### Data collection

Data were collected through focused group discussions where the oral opinions of the interviewees were recorded. The reason for choosing this method was its ability and power to identify and explore opinions, beliefs, and attitudes of experts regarding specific issues [26]. A focus group discussion questionnaire guide was used to conduct the sessions. First, using the related literature and based on the study objectives, a group discussion questionnaire guide was compiled. Then the validity of this guide was evaluated by conducting a pilot study (focus group discussion with 5 managers and employees as well as an individual interview) and the necessary corrections were made to the guide.

Participation in the sessions was completely optional, and after obtaining written informed consent from the participants who were already briefed on the objectives of the study, the group discussion sessions were held in the meeting hall of the Deputy of Health. Seven group discussion sessions were held with the participation of managers, officials and staff. Two sessions with the managers and officials of the deputy of health of the faculty (the number of participants in each session was between 8 and 10), 4 sessions with the experts (the number of participants in each session was between 10 and 15) and one session was held with the participation of 5 managers and 10 experts in order to summarize the findings. The total number of participants in all sessions was 60. Each session lasted from 60 to 90 minutes. The main focus of the sessions was on how to provide modern health services to middle-aged men in the health bases, health posts, and comprehensive health service centers and on the associated challenges. Since data collection was during the spread of the Covid-19 disease, safety precautions such as maintaining social distancing and using masks were observed in the meeting hall of the Deputy of Health. The sessions started with simple questions and the participants were given some time to reflect on the answers. After the discussion progressed a bit, more original and challenging questions were raised: “What challenges do men face while going to the centers?“ “What challenges do men face while going to health centers and bases to receive health care?“ What are the challenges of the middle-aged care program for men?“ At the end of the discussion, the final questions elicited concluding remarks. The data were collected by note taking. The conversations were recorded for a better evaluation of the sessions.

### Data analysis

Data analysis was done manually using the conventional content analysis method with data reduction. Content analysis is a method of explaining concepts through examination of people’s experiences and viewpoints as well as the analysis of common factors between these viewpoints [31]. Based on this method, analysis and interpretation are done in 5 steps, including the researcher familiarity with the data, generation of initial code for concepts, identification of themes, reviewing of the themes, and numbering of the themes [30].

Immediately after each group session, the tapes were transcribed verbatim. The transcribed text was reviewed several times and compared with the notes of two members of the research team (SME and TR). Comprehensible concepts and semantic units were extracted, summarized, and coded. Similar codes were grouped according to a common concept in order to form subcategories. Each category was given a label representing the codes under that category. By comparing the relationships, concepts, contradictions and opinions observed, the themes were extracted from the findings.

### Scientific trustworthiness of the results

**Fig. 2:**
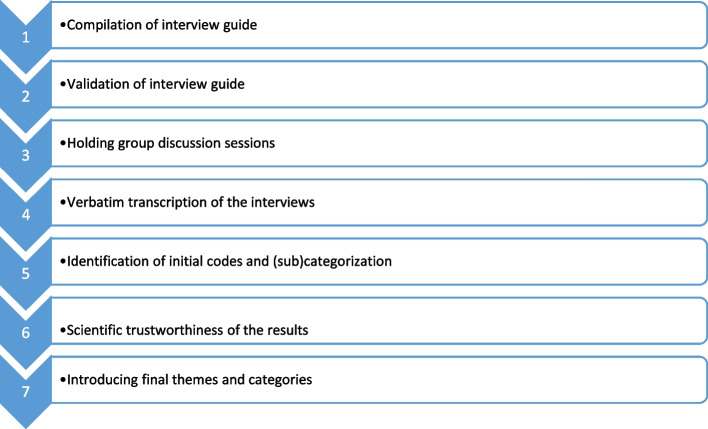
Study process

Lincoln and Cuba’s four-criteria, namely credibility, dependability, confirmability, and transferability were used to assess the trustworthiness of the results [32]. As far as credibility was concerned, we continued sampling until data saturation. After each focus group discussion session, the recorded conversations were transcribed and given to the participants to confirm their authenticity. After the reduction process, the transcription was given once again to the participants for approval. To ensure transferability of data, we comprehensively described the topic, the participants, data collection, and data analysis. To ensure the dependability of the data, coding was done simultaneously by (SME and TR). Then comparisons were made, disagreements were resolved, and consensus was achieved by all the research team members. Investigator triangulation was performed in order to enhance confirmability of the findings [33]. Finally, after the collected data were categorized and checked with the participants, to receive final confirmation, we presented the data in a seventh focus group session [34]. Study process is illustrated in Fig. 2.

## Results

The mean of the participants’ age was 34.1 ± 7.2 years, with most participants being over 30 years old. Most of the participants in this study were women. The average management experience of the participants was 10.5 ± 5.1 years. More than half of the managers had less than 10 years of management experience. Urban community health workers had the highest rate of participation in this study. The interviewees’ average work experience was 11.0 ± 6.6 years. Most participants had less than 10 years of work experience (Table 1).

**Table 1:** Demographic information of the interviewees

Variable	Levels	Frequency (Percentage)	Variable	Levels	Frequency (Percentage)
Age (year)	20–30	18(30)	Management experience (year)	1–10	12 (54,4)
	31–40	30 (50)		11–20	8 (36,4)
	41–50	12 (20)		21–30	2 (9,2)
Sex	Male	12 (20)	Work experience (year)	1–10	30 (50)
	Female	48 (80)			
Education	High school Diploma	10 (16,7)			
	Associate degree	8 (13, 3)		11 − 10	24 (40)
	Bachelor’s degree	20 (33,3)			
	Master’s degree	16 (26,7)		21–30	6 (10)
	General physician	5 (8,3)			
	PhD	1 (1,7)			
Occupation	General physician	5(8,3)	Workplace	Headquarters	24 (40)
	Health Services Manager	1(1,7)		Urban CHSCs	4 (6,7)
	Urban community health worker	36(60)			
	Rural community health worker	14 (23,4)		HBs	6 (10)
	Nutritionist	2 (3,3)		Rural CHSCs	14 (23,3)
				HPs	12 (20)
	Mental health worker	2 (3,3)			

**Fig. 3:**
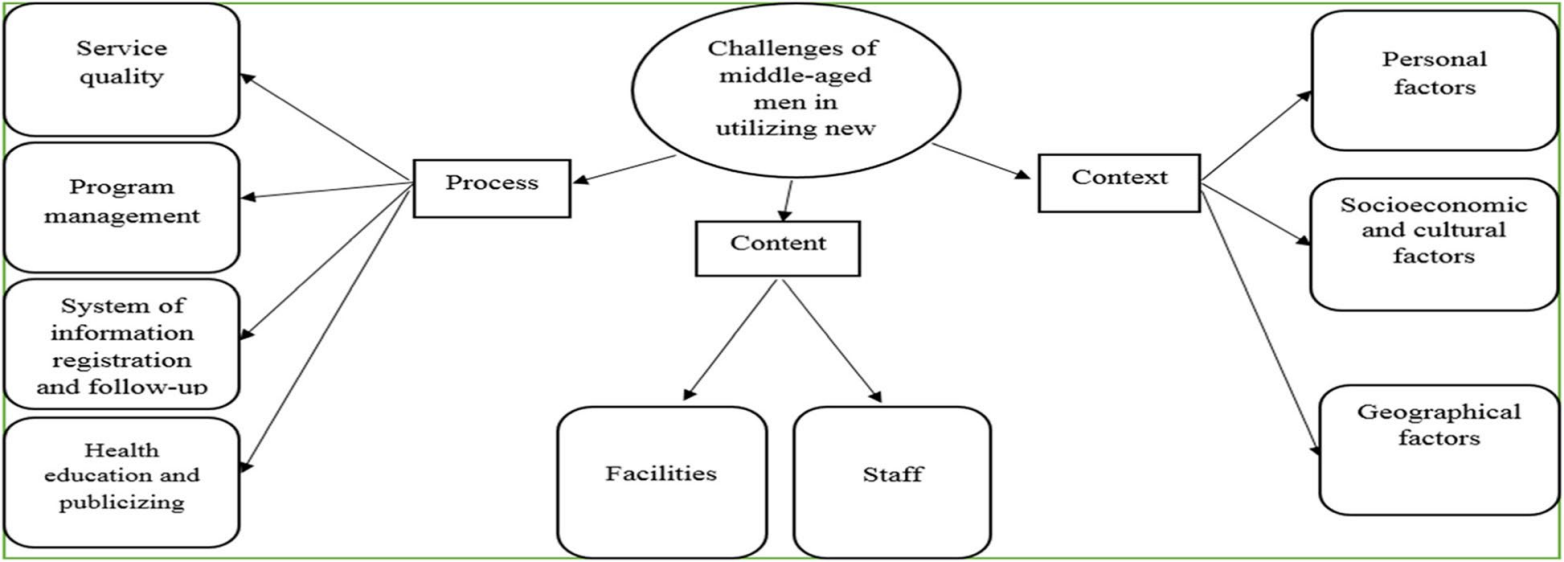
Challenges of middle-aged men in receiving modern health services

Findings from group discussion were organized into 35 codes, 9 categories, and 3 main themes (Fig. 3). The main themes included Context, Content, and Process. The Context consisted of 13 codes and 3 categories (personal, socioeconomic and cultural, and geographical factors). The Content included 9 codes and 2 categories (staff and facilities). The Process consisted of 13 codes and 4 categories (service quality, program management, information registration and follow-up, health education and publicizing) (Table 2).

**Table 2:** Context-related challenges of middle-aged men in utilizing new health services

Category	Codes	Similar meanings
**Personal factors**	Not having enough time to get health services due to job-related reasons	• The working hours of CHSCs, HBs and HPs are synchronous with the men’s working hours • The middle age is one of the active and working age groups
	Macho pride	• Failure to refer due to feeling strong
	Men’s fear of the disclosure of their secret illness and personal information	• Men’s lack of trust in the health information registration system • Men’s fear of the possibility of a disease • Men’s high sensitivity to disclosure of information related to their illness
	Men’s preference to see specialists	• Men’s reluctance to see general practitioners • Men’s reluctance to refer to health workers
**Socioeconomic and cultural factors**	Economic problems and men’s responsibilities regarding livelihood and family care	• National economic problems and inflation • Men’s traditional responsibility for family livelihood • Low income and the staggering cost of living
	Health not as a priority in men	• Arbitrary use of painkillers by men to relieve pain and treat illness • Men’s ignorance of the alarming signs of diseases • Inadequate literacy of men regarding the importance of health and wellbeing
	Men’s refusal to refer to female urban community health workers	• Lack of male urban community health workers in the health system • Imbalanced health care personnel by gender at service delivery points • Shame and modesty in religious men to see a female urban community health worker
	Addiction and history of imprisonment in men	• Addicted men’s refusal to receive health services • Inability of addicted men’s wives in persuading their husbands to receive services due to the women’s fear of being beaten
	Ethnic and tribal problems in rural areas	• Some men’s refusal to go to HPs due to ethnic and tribal disputes
	False beliefs	• Extreme belief of Iranian men in herbal drugs • Men’s disbelief in new health services
**Geographical factors**	Long distance between home or workplace and health centers	• Men’s lack of permanent residence due to job-related reasons • Inadequate physical access to services in rural areas
	Hot climate of south of Iran	• Men’s failure to refer to health centers due to the extremely hot weather in south of Iran
	Improper location of some health service centers	• Not using GIS in locating CHSCs, HBs and HPs for providing comprehensive health services in rural and urban areas

### Context-related factors

Contextual factors are personal, socioeconomic, and cultural categories that are effective in seeking health services. They are mainly related to sex, age group, level of literacy, employment status, type of job and work shift, economic and cultural factors, urban or rural status, and climate [35–37].

#### Personal factors

These are factors that originate from men’s personal behaviors and norms [37]. According to the research findings, the category of personal factors included the following subcategories: Not having enough time to get services due to job-related reasons, Macho pride, Men’s fear of the disclosure of their secret illness and personal information, and Men’s preference to see specialists.

The participants believed that a number of factors, including personal factors and attitudes, prevent middle-aged men from visiting health centers. Occupational factors were cited as the most important barrier to using health care services in this age group:

“The center’s working hours are when men are at work, so because men are busy at work, they can’t go to the centers in the morning.” (P7).

With respect to men’s fear of disclosure of information about their secret illness, another participant said:

“Men are afraid of having a disease in their body that makes them feel powerless and weak in taking care of the family.” (P33).

In this regard, one of the managers added:

“One of the problems is that people do not like their information to be recorded. We have to brief men that their information is confidential. Men are very sensitive about their disease information being recorded somewhere.” (P2).

According to research findings, personal factors were one of important reasons for the non-referral of middle-aged men.

#### Socioeconomic and cultural factors

Some of the issues that prevent men from using health services are rooted in economic, social, and cultural contexts [9]. The sub-categories of this category include Economic problems and men’s responsibilities regarding livelihood and family care, Health not as a priority in men, Men’s refusal to refer to female urban community health workers, Addiction and history of imprisonment in men, Ethnic and tribal problems in rural areas, and False beliefs.

With respect to economic problems and men’s responsibilities regarding livelihood and family care and health not as a priority in men, one of the interviewees stated:

“Economic problems make men give priority to their work and prefer to go to their work rather than the health center.” (P3).

Some interviewees believed that middle-aged men did not believe in new health services and were more willing to use traditional medicine to treat their illnesses.

“Men often believe in traditional medicine. When they get sick, they are more likely to use drugs from herbalists and do not believe in new medicine.” (P40).

Given the fact that most of the health workers in the service centers are female, the participants believed that the existence of religious beliefs in the Iranian society causes middle-aged men to refrain from referring to female urban community health workers:

“One of the things I feel prevents men from going to the centers is that we do not have male community health workers. I think this is very, very important. And this is important in two ways. After all, most of the population here is Muslim and they have a set of beliefs; and a set of restrictions and boundaries must be observed between men and women. This is true for both men and women.” (P4).

Another participant cited ethnic and tribal problems in rural areas as an obstacle to receiving new health services:

“The existence of some tribal and ethnic problems prevents men from going to the centers. A gentleman came and took his notebook. I told him to get a letter from the rural health worker, but he said ‘I won’t do it because we have broken up with their family’” (P15).

The impact of economic, social and cultural problems in the field of health is one of the most important issues in receiving health services from the perspective of participants.

#### Geographical factors

Geographical factors are among the factors that can facilitate access to health care [38]. Geographical issues such as the long distance between the living or working place to service centers, hot weather in the south of Iran, and inadequate location of some service centers were among the reasons that the studied men were reluctant to refer to these centers. Regarding the long distance between home or work to service centers in rural areas, one participant said, “The distance from the health post to the health center is very long. The health worker refers someone to the health center, but when that person gets there, the doctor has already gone to another village, so they can’t see the doctor, and since they are not a resident of that area and they work elsewhere, they cannot get the service they need.“ (P9) Another participant pointed out that the inappropriate location of some service centers is the reason why middle-aged men do not come to receive new health services. This participant recounted, “A few days ago, two clients who were neighbors came here. One of them was under the coverage of by our base, but the other was related to another base. The former said, ‘I will not come here anymore because my neighbor goes to another base but I have to come here while we both live in the same alley’ (P39) The findings of the study showed that ignoring geographical factors is effective in preventing middle-aged men from referring to service centers.

### Content-related factors

Content-related factors include personal factors and interpersonal relationships in the organization. They constitute the behavioral norms, informal communication, specific interconnected patterns, and the main (material and immaterial) content of the organization and play a significant role in encouraging individuals to receive services [39].

Content challenges of middle-aged men in utilizing new health service are listed in Table 3.

**Table 3:** Content challenges of middle-aged men in utilizing new health service

Category	Codes	Similar meanings
**Staff**	High workload	• Failure in following up the men’s health program due to the high workload of PHC workers
	Lack of manpower in service centers	• Lack of manpower to follow up the men’s health program
	Inadequate payment of salaries and welfare allowances	• Failure to pay employees’ salaries on time • Failure to pay overtime and merit pay to private urban community health workers • Employee dissatisfaction with support services
	Lack of job stability in urban community health workers with a contract employment	• Lack of employment security in private urban community health workers
	No path to promotion and career advancement	• Lack of a system for career advancement and promotion for private urban community health workers • Differences in job specifications and payments of the staff in health division and those in treatment division
	Disrespect to community health workers by their superiors	• Mistreatment, disrespect and threats of dismissal by officials • Not appreciating the work of community health workers by their superiors
	Humdrum of work routine and job burnout in experienced manpower	• Mismatch between the staff profession and the new needs and services • Time consuming documentation in systems
**Facilities**	Inadequate and inappropriate physical space	• Lack of private physical space to provide services to middle-aged men • Lack of appropriate physical space for education
	Inadequate and inappropriate equipment	• Lack of cooling and heating equipment in CHSCs, HBs and HPs • Weak support for CHSCs, HBs and HPs

#### Staff

The use of health technologies in the management and provision of services in order to improve the level of health is the responsibility of the health system staff. Paying attention to human resources as the most vital element of the organization’s success in organizations providing health services is always considered a managerial priority [40].

Participants identified staffing topics and issues as barriers to providing new health services to middle-aged men. High workload, lack of manpower in service centers, inadequate salaries and welfare facilities, lack of employment security in contract private urban community health workers, lack of career promotion, disrespect to community health workers by their superiors, a humdrum life, and burnout of highly experienced forces were among these barriers. Concerning high workload, one of the staff said:

“We are only two [colleagues] working in the center and our workload is very high. If my colleague goes on leave, I will be crushed under the workload.” (P18).

Another staff participant commented on the humdrum life and exhaustion of the highly experienced forces:

“We asked the staff to register their work in the system and this has added to their daily routine, so we took a part of their time in which they could provide useful services. Now, apart from the motivation of the staff, work has become humdrum for them.” (P23).

As far as promotion path and career advancement was concerned, one of the staff participants said:

“In the Education Office, those who continue their education will acquire a PhD or a master’s degree but with the same category of work, but [in the Health Ministry] one becomes a doctor and receives 100 to 150 million IRR a month. In health sector, there is a difference between a job and the title of payments, so they compare themselves with a doctor. One is a doctor and the other is a community health worker.” (P14).

Our findings showed that staffing problems has a negative impact on the process of providing services to clients.

#### Facilities

A community’s benefit from health services and medical facilities is one of the main indicators of development. One of the most important problems in providing healthcare services in third world countries is lack of healthcare facilities [41]. Some of the staff participants believed that there is a series of problems in relation to infrastructure and issues related to facilities, which prevent men from using new health services. Inadequate and insufficient physical space for education and delivery of services, and inadequate and insufficient equipment in health corresponding to the needs of men were among the influential factors in this respect. According to the interviewees, the lack of respect for the patient’s privacy in the service centers had made middle-aged men reluctant to receive new health services. One of the interviewees pointed to the inadequate physical space for education and delivery of services:

“There is no private counseling room for men in the centers, so men cannot have enough confidence and speak freely. (P23)

Another maintained:

“We do not have measurement equipment in the center, and the buildings are very old and worn out, and there is no suitable place for clients. There are educational media, but they are not used due to the lack of physical space.” (P11).

The interviewees believed that lack of physical space and equipment contributed to failure in delivery of services to clients, especially middle-aged men.

### Process-related factors

Process-related factors were among the controversial challenges of middle-aged men to receive modern health services. These factors include the method of execution and implementation of the program. In the process of implementing a program, appropriate empowering processes should be used in each step so that the acceptance of the program is met with less resistance [42] (Table 4).

**Table 4:** Process challenges of middle-aged men in utilizing new health services

Category	Codes	Similar meanings
**Service quality**	Poor communication skills while interacting with clients	• Staff’s use of vague and incomprehensible terms • Poor counseling • Staff’s lack of sufficient skills to hold group education
	Incomplete provision of middle-aged service package	• Absence of a physician at the time of services delivery • Elimination of services required by men from service packages such as kidney and urinary tract services
	Poor hygienic conditions of the health service centers	• Ignoring beautification of service centers • Not observing hygiene in service centers
**Program management**	Poor referral system	• Inadequate follow-up of care within the referral system • Lack of proper division of labor in the referral system • Shortage of doctors and experts
	Low priority of men’s health program within the health system	• Low priority of men’s services in the service registration system (46)
	Shortage of male urban community health workers in service centers	• Not hiring male urban community health workers in service centers
	Inadequate utilization of the capacities and participation of other sectors	• Not using the potential of Dehyari (Rural Governor Offices) • Not using the potential of factories, offices and clubs
	Interest in the quantity of activities	• Managers’ interest in the quantity of the activities of health workers • Health workers’ haste in providing more but incomplete services
**System of information registration and follow-up**	Frequent disconnections in the Internet and intranet network	• Poor coverage of Internet and intranet networks in CHSCs, HBs and HPs
	Systems problems	• Impracticality of inviting middle-aged people through sending SMS in the systems (46) • Incomplete provision of reports on men’s health status in SIB system
	No active house-to-house follow up by community health workers	• Unavailability of a vehicle for community health workers to do service follow-ups • Community health workers’ insecurity and being disrespected during follow-ups
**Health education and publicizing**	Inadequate publicizing and poor health education	• Inadequate publicizing of the available services • System inefficiency in holding training courses for the personnel
	No active presence of the health system in cyberspace for publicizing	• Not using the competitive advantages of cyberspace to attract people • Insufficient training for community health workers

#### Service quality

There is not much agreement on the definition of quality. Service quality is known as an important criterion for the performance of health service organizations. Health organizations are looking for meeting customer demand and achieving continuous quality [43].

Most participants were of the opinion that the low quality of services in CHSCs, HBs and HPs is a barrier to the delivery of new health services to middle-aged men. According to our findings, the staff’s poor communication skills, incomplete provision of middle-aged service package, and the inadequate hygienic measures in service centers were identified as the most important reasons in this respect. Regarding the staff’s poor communication skills, one participant said:

“The words and phrases used by community health workers may be comprehensible to the educated community health workers themselves, but not to some lay men with relatively low literacy. For example, when talking about prostate or testicular disease, this should be comprehensible to illiterate men and tailored to their language.”(P19).

Regarding poor counseling skills, another participant said:

“To care for the middle-aged, the staff should be more skilled and should have a higher power of counseling.” (P32).

As far as the incomplete provision of middle-aged service package was concerned, one of the managers commented:

“We did not provide the complete services that we already introduced. One of the problems is that a client comes to the campaign to have their blood pressure measured, but the doctor is not available, and this is despite the fact that we have already emphasized that they [clients] should attend the center at this particular time to get their blood pressure measured, and the visit will be free of any charge.” (P2).

Another manager maintained in this respect:

“One of the challenges is about kidney and urinary tract diseases because men often suffer from kidney and urinary tract diseases. This plan was a very good plan. It was launched and implemented for a while, but then it was eliminated.” (P5).

From the perspective of primary health workers, the quality of services was one of the important issues in the number of men referring to service centers.

#### Program management

The issue of management in providing health services was emphasized by the participants in the study. In order to prevent disease and reduce premature mortality, necessary plans should be developed and implemented using scientific standards and according to the conditions of each region. Program management includes important concepts such as planning, organization, coordination and control [44].

Most participants believed that improper program management in CHSCs, HBs and HPs was a serious barrier preventing middle-aged men from referring to service centers. According to the participants, poor referral system, no priority of men’s health program within the health system, shortage of male urban community health workers in service centers, inadequate utilization of the capacities and collaboration of other sectors, and interest in the quantity of activities were among the most important problems in this respect. According to one of health center staff,

“There is a big gap between a visit and a doctor’s referral; or when we tell someone to come here to see a doctor, they will come but the doctor is not at the center; the next time they won’t come for different reasons, and there is no follow up of the referrals.” (P23).

Regarding the low priority of the men’s health program within the Iranian health system, another participant said:

“One of the problems with the SIB system (Farsi acronym of The Integrated Electronic Health System that was implemented and launched in Iran in order to delivery of health services in the form of health system reform programs and projects. All information about households, the type of health care services required in CHSCs, HBs and HPs are entered and recorded in this system) is that the men’s health program is not a priority in this service system. This system gives weight to different services. It gives 3 to one service, for example, but gives 1.5 to services for middle-aged men, and then it gives priority to pregnant women.” (P14).

The interviewees in this study believed that neglecting recruitment of male urban community health workers in service centers contributed to limited delivery of services to men:

“We do not have a male community health workers here. The Health Transformation Plan was per se to employ a male community health worker in each center, but this was not implemented.” (P33).

In relation to the managers’ interest in the quantity of activities, another participant said:

“When we tell health care workers that you have to provide a lot of services today, this reduces the quality. Well, our colleague did not give enough information to the client. If a male client comes and we cannot provide the services perfectly, he will go and negatively publicize this, saying ‘I went there and they did nothing for me’, and this reduces the number of referrals.” (P24).

According to the research findings, problems related to program management had an indirect effect on middle-aged men’s refusal to visit CHSCs, HBs and HPs.

#### System of information registration and follow-up

This means the existence of an information system aimed at providing health-therapeutic services [25]. Poor system of information registration and follow-up was identified as another reason for middle-aged men’s refusal to visit health service centers. Findings from the present study showed that the frequent disconnections in the Internet and intranet networks, problems related to systems, and lack of active house-to-house follow-up by community health workers due to insecurity were identified as the main barriers in this respect.

“The Internet connection constantly goes off and on. Sometimes when we want to deliver a service to people, we wait so long for the Internet to reconnect that most of the clients decide to forget about receiving the service; in general, we always have these problems with the system.” (P22).

“We, the community health workers, are mostly women. We do not have a proper vehicle and we are afraid to go into the alleys for house-to-house follow up of our services,” said one of the urban community health workers with regard to absence of active house-to-house follow-up done by community health workers. “We are not treated well. We are not safe here.” (P18).

#### Health education and publicizing

This means the role of health staff and mass media in informing people and introducing services [25].

According to the participants, inadequate publicizing and poor health education along with no active presence of the health system in cyberspace for publicizing health were among the most important obstacles in this regard.

As far as inadequate publicizing was concerned, one of the participants said:

“As soon as we delivery oral health services in the centers, many people say that they had not been informed that such a service is provided. If information about the services offered in the centers is provided correctly, the number of referrals will increase. ” (P13).

“We need to receive more education and more retraining courses so that we can be accountable and gain the trust of the people. Therefore, if someone goes to a health care center, that will benefit them.” (P32).

Some managers’ viewpoint was more focused on the organization’s weaknesses in being actively present in cyberspace for advertising. One of the managers said:

“The active presence and participation of the health system in the use of mass media and the Internet can be very effective. We must go there and play a more prominent role. What is at stake here is our competitive advantage. We have to work on competitive advantages, be more attractive, and attract more people.” (P1).

## Discussion

The aim of this study was to examine the challenges of middle-aged men in utilizing health services from the perspective of primary health workers. The results of the present study showed that in order to utilize health services, the middle-aged men in Iran face three types of barriers related to context, content, and process.

Findings of the study showed that the context-related barriers include personal, socioeconomic and cultural, and geographical factors that create challenges for middle-aged men in receiving health care. From most of the participants’ point of view, personal barriers were recognized as one of the most important reasons middle-aged men did not refer to use new health services. The following factors have been cited in the literature as the ones contributing to men’s failure in referring to receive health services: the issue of macho pride, socio-economic problems, not feeling the need for care, lack of awareness of the need for care, ethnic and racial differences, men’s concern about confidentiality, views related to gender norms, religion and spirituality, addiction, and (mis)beliefs and false beliefs have been mentioned in the non-referral of men to [4, 25, 45, 46]. One of the barriers that health workers cited as a factor in preventing middle-aged men from seeking new health services was that the working hours of the health care facilities were concurrent with the working hours of these men. In their study on the reasons of men’s reluctance to participate in family planning programs, Fathnezhad Kazemi et al. [46] and Hosseini et al. [25] concluded that the working hours of the centers did not match the men’s free time. Ramezani Tehrani et al. [9] conducted a qualitative study in Tehran and proposed that having multiple jobs and not having enough time is two of the most important health barriers that prevent men from thinking of their health as a priority, which is consistent with the results of the present study. Craig et et al. [4] in another qualitative study also identified the issue of machoism in men as one of the most important reasons for not using health services. Regarding the preference of men to refer to specialists, Tudiver Talbot [24] and Rasouli et al. [47] obtained results similar to the statements of the participants of the present study. Hosseini et al.‘s study [25]also showed that Iranian men tend to refer to specialists to receive care. In the meantime, socioeconomic and cultural factors were other important factors that played a role in preventing men from referring to health centers. In line with this study, Ramezani Tehrani et al. [9] identified economic and socio-cultural problems as factors that prevent men from considering their health as a priority. These included factors such as social damage caused by addiction, lack of preventive culture, and ignoring the importance of health. In Hosseini et al.‘s study in Bojnord [25], no significant relationship was found between the economic status and Iranian men’s non-referral to health centers and health bases, which is inconsistent with the results of the present study. This discrepancy in results can be due to the difference in research methods, population and setting. In order to remove obstacles related to contextual factors, it is necessary to devise a comprehensive program that facilitates the provision of modern health services to this age group according to their type of job and working hours and by maintaining their privacy and confidentiality at work. Measures such as increasing the working hours of health centers, providing mobile services, and providing home and telemedicine services can have a great impact on improving the condition of middle-aged men.

Another group of challenges for middle-aged men in receiving services are content-related factors. This theme includes factors related to staff and facilities and can play a prominent role in receiving care by middle-aged men. Consistent with the results of the present study, Allahyari et al. [48] found that factors such as high workload of health care workers, appreciation and respect, salaries and benefits, and job stability were among the challenges of health workers which affected their provision of quality health care to clients. Heidari et al. [49] also reported that health centers are inadequate in terms of their manpower, physical space and equipment. The shortage of male urban community health workers in service centers of Shoushtar was also one of the challenges mentioned in this respect. The results of a study by Tudiver and Talbot [24] conducted in Toronto showed that shortage of male manpower in service delivery units is one of the reasons for men’s limited access to health care system, which is consistent with the results of this study. Another challenge middle-aged men face in receiving health services was the inappropriate physical environment and insufficient equipment, which have also been mentioned in some studies [25]. Increasing the number of community health workers, reducing work stress in the staff, creating respectful working conditions, and procuring new and high-quality equipment can have a significant impact on eliminating this type of barriers.

The third type of challenges of middle-aged men in utilizing new health services according to the present study were process-related factors. In order for the better management and improvement of the quality of the new health service program for the middle-aged, more efforts should be made in terms of processes related to education and publicizing, referral system, and information registration system.

Communication skills play an important role in education and information. In Hosseini et al.‘s study [25], the poor communication skills of the staff and the incomplete provision of services negatively affected the rate of patient visits. The results of other studies in Iran [50, 51] also indicated that the communication skills among medical professionals is not desirable and it seems that this weakness can prevent effective communication with clients and providing optimal services. According to the interviewees, inappropriate publicizing and advertising was the cause of middle-aged men’s not knowing about the existence of services in centers and bases. This has even led to the assumption that the focus of primary health care is only on the needs of women and children [25]. The participants believed that the weakness in the referral system was another reason for middle-aged men’s failure in receiving services. Various studies addressing the referral system [52–54] have also emphasized the inappropriate status of the referral system program within the health system, which is consistent with the participants’ statements. The greatest inconsistency is observed between primary and secondary service provision. Despite the implementation of the family physician program and the referral system, these problems have not been resolved. It seems that it is necessary to revise this program based on the conditions of each province.

Another process-related challenges mentioned by the participants were the inadequate system of information registration and follow-up. In Iran’s health system, several systems are used to record health information in different provinces. In Shoushtar, the Integrated Health System (the Persian acronym of which is SIB meaning “apple”) includes the individual’s health care information. All information related to households, the type of health care services needed in community health centers and health bases, and health posts are entered and recorded in this system. Some studies have reported that the frequent disconnection of the Internet and intranet networks, incomplete reports, the time-consuming nature of working with the system waste the clients’ time and make it practically impossible to deal with them in service centers [55, 56]. Electronic registration of services in Iran requires the development and improvement of the necessary hardware and software infrastructure. For the successful implementation of the leveling of services, the family physician program, and the referral system, there needs to be more cooperation and coordination between the health and treatment sectors (providing primary, secondary and tertiary services). Improving human resources and infrastructure can play a significant role in improving process challenges associated with providing services to the middle-aged.

### Limitations

This study deals with Challenges of middle-aged men in utilizing new health services from primary health care providers’ perspective, and it is necessary to examine the views and opinions of service recipients in a similar qualitative study. The conflict of interests of managers and employees participating in the study in expressing challenges and solutions was another limitation of this research that may have affected the results.

## Conclusion

Factors related to context, content and process were identified as determining factors in providing modern health services to the middle-aged. In order to increase middle-aged men’s benefit from the aforementioned services, contextual factors can play an important role in removing time and space restrictions. By the same token, content factors can improve the quality and quantity of human resources, the working conditions of health care providers, and the facilities of service providers. Finally, by proper management of leveling of service provision, family physician program, and the referral system, process factors can promote men’s referrals to receive services and improve their care. Similar future studies may focus on ways to increase men’s visits to health centers.

## Supplementary Information

**Additional file 1: Table a1.** Some relevant studies about middle-aged men health services in Iran and the world.

## Data Availability

All data generated or analyzed during this study are included in this published article.
