# Supplementary material for: Challenges of middle-aged men in utilizing new health services from primary health care providers' perspective: a qualitative study
Source: BMC Prim Care. 2022 Dec 7;23:318. doi: 10.1186/s12875-022-01933-2 (PMC9730688; doi:10.1186/s12875-022-01933-2)
Supplement: Supplementary file 1 — Additional file 1: Table a1. Some relevant studies about middle-aged men health services in Iran and the world. [file 12875_2022_1933_MOESM1_ESM.docx]

Appendix 1

Table a1: Some relevant studies about middle-aged men health services in Iran and the world

| Row | first author | Publication date | Type of study | Target group/Purpose of the study | Tools | Findings |
| --- | --- | --- | --- | --- | --- | --- |
| 1 | Hosseini Z ... (1) | 2020 | Descriptive cross-sectional | Men aged above 18 | Researcher made questionnaire | Inadequate physical conditions of health centers and lack of trust in health personnel were recognized as the most important reasons for men's failure to visit community health centers. |
| 2 | Ramezani Tehrani F ...(2) | 2010 | Qualitative | Identification of the most important factors inhibiting mental health in men of different ages | Group discussion | The most important factors preventing the prioritization of health in men include economic, cultural, social, family factors, lack of social security, weak macro managerial and environmental factors. |
| 3 | Ramezani Tehrani F ...(3) | 2009 | Qualitative | Identification of barriers to healthy nutrition | Group discussion | Barriers to healthy nutrition were explained in five categories: 4"improper education and culture", "behavioral patterns", "lack of access", "role of women" and "type of taste and personal interest". |
| 4 | Rostamian A.A.R ...(4) | 2001 | Qualitative | Identification of the causes of men's failure to participate in the family planning program | Group discussion | Inefficient advertising, insufficient information, the mismatch between the working hours of the centers and the free time of men, the lack of a suitable space for face-to-face counseling for men, and the lack of male counselors have contributed to the severity of the non-participation of men. |
| 5 | David L ...(5) | 2013 | Review | Providing solutions for the health of young men based on the goals of the Center for Disease Control and Prevention | Review of literature | Employing male health care providers, creating time and space for counseling, screening for tobacco use and diabetes, reducing mortality, violence, and unintentional injuries, and paying attention to sexual and reproductive health, mental health and substance abuse are the most important suggestions of this study for achieving the goals of the Center for Disease Control and Prevention. |
| 6 | Arruda GO ... (6) | 2016 | Cross-sectional | Assessment and identification of factors related to the use of health services in men aged 20-59 | questionnaire | Hospitalization is the most common reason for using medical services |
| 7 | Craig F ...(7) | 2008 | Review | A review of studies on men's health and macho pride | Review of literature | Revisiting the structure and processes of providing health care to men and its components can be effective in men's use of services. |
| 8 | Galdas PM... (8) | 2005 | Review | Identifying driving behaviors to help men's health in databases | Review of literature | A healthy lifestyle plays an important role in men's health and reducing male mortality. Gender, traditional male behaviors, and socioeconomic status affect men's health. Reconsidering the structure and processes of how men's health care is delivered can help engage men in health-related activities. |

1. Hosseini Z, Ghaffari M, Rakhshanderou S, Jafari Y, Shoraka HR. Investigating the Reasons for Not Referring Men to Health Centers to Receive Health Services. Journal of North Khorasan University of Medical Sciences. 2022;14(1):29-37.
2. Ramezani Tehrani F, Amiri P, Simbar M, Rostami Dovom M, Azizi F. Do Men Consider Health as a Priority? A Qualitative Study. Hakim Health Systems research journal. 2011;13(4):241.
3. Ramezani Tehrani F, Maryam Farahmand, Parisa Amiri, Arash Ghanbarian, Fereidoun Azizi. Healthy nutrition barriers: perception of adults male. Health Monitor Journal of the Iranian Institute for Health Sciences Research. 2012;11(5):725-35.
4. [Rostamian A.A.R.](https://www.sid.ir/en/journal/SearchPaper.aspx?writer=32991), [Heydari Gholam Reza](https://www.sid.ir/en/journal/SearchPaper.aspx?writer=7859), [Zafarmand M.H.](https://www.sid.ir/en/journal/SearchPaper.aspx?writer=44976), [Asaadi K.](https://www.sid.ir/en/journal/SearchPaper.aspx?writer=169331), [Bar Ghandan R.](https://www.sid.ir/en/journal/SearchPaper.aspx?writer=169332), [Dianat Mahin](https://www.sid.ir/en/journal/SearchPaper.aspx?writer=158421), [Bahtouei M.](https://www.sid.ir/en/journal/SearchPaper.aspx?writer=24794) Men's Disinclination In Family Planning In Bushehr Port Using Focus Group Discussion. [Iranian South Medical Journal (Ismj)](https://www.sid.ir/en/journal/JournalList.aspx?ID=4042)   [February 2002 , Volume 4 , Number 2](https://www.sid.ir/en/journal/JournalListPaper.aspx?ID=49534); Page(S) 142 To 149.
5. BELL, David L.; BRELAND, David J.; OTT, Mary A. Adolescent and young adult male health: a review. *Pediatrics*, 2013, 132.3: 535-546.‏
6. ARRUDA, Guilherme Oliveira de; MARCON, Sonia Silva. Survey on the use of health services by adult men: prevalence rates and associated factors. *Revista Latino-Americana de Enfermagem*, 2016, 24.‏
7. Garfield Cf, Isacco A, Rogers Te. A Review Of Men's Health And Masculinity. American Journal Of Lifestyle Medicine. 2008 Nov;2(6):474-87.
8. Galdas Pm, Cheater F, Marshall P. Men And Health Help‐Seeking Behaviour: Literature Review. Journal Of Advanced Nursing. 2005 Mar;49(6):616-23.
